# An Architectural Multi-Agent System for a Pavement Monitoring System with Pothole Recognition in UAV Images

**DOI:** 10.3390/s20216205

**Published:** 2020-10-30

**Authors:** Luís Augusto Silva, Héctor Sanchez San Blas, David Peral García, André Sales Mendes, Gabriel Villarubia González

**Affiliations:** 1 Expert Systems and Applications Lab—ESALAB, Faculty of Science, University of Salamanca, Plaza de los Caídos s/n, 37008 Salamanca, Spain; hectorsanchezsanblas@usal.es (H.S.S.B.); daveral@usal.es (D.P.G.); andremendes@usal.es (A.S.M.); 2 Laboratory of Embedded and Distribution Systems, University of Vale do Itajaí, Rua Uruguai 458, C.P. 360, Itajaí 88302-901, Brazil

**Keywords:** smart applications, drones, YOLOv4, crack detection, virtual organizations of agents

## Abstract

In recent years, maintenance work on public transport routes has drastically decreased in many countries due to difficult economic situations. The various studies that have been conducted by groups of drivers and groups related to road safety concluded that accidents are increasing due to the poor conditions of road surfaces, even affecting the condition of vehicles through costly breakdowns. Currently, the processes of detecting any type of damage to a road are carried out manually or are based on the use of a road vehicle, which incurs a high labor cost. To solve this problem, many research centers are investigating image processing techniques to identify poor-condition road areas using deep learning algorithms. The main objective of this work is to design of a distributed platform that allows the detection of damage to transport routes using drones and to provide the results of the most important classifiers. A case study is presented using a multi-agent system based on PANGEA that coordinates the different parts of the architecture using techniques based on ubiquitous computing. The results obtained by means of the customization of the You Only Look Once (YOLO) v4 classifier are promising, reaching an accuracy of more than 95%. The images used have been published in a dataset for use by the scientific community.

## 1. Introduction

Road safety is key to reducing the number of road accidents. The main solutions often focus on reducing speed limits, but road conditions are also important in the increase in road accidents. A recent study carried out by the AEC stated that the poor condition of roads is the main cause of 94% of accidents [1]. According to this organization, 1 out of every 13 km of the Spanish road network shows significant deterioration in more than 50% of the surface of the pavement as a result of accumulating potholes, ruts, and cracks. This damage affects vehicle safety, creating a less stable and safe driving environment. The report [1] also shows that Spain is failing to maintain its roads, and describes the condition of the asphalt as very poor. This translates into an increased risk of an accident in the worst-case scenario, but can also lead to vehicle breakdowns. To minimize the risks of a possible accident or decrease the possibility of a breakdown, it is vitally important to investigate and develop techniques that can be used to improve the road infrastructure. The difficult worldwide economic situation has repercussions for the state of roads, making maintenance and improvement of transport routes lower priorities.

To determine which roads require some type of improvement or maintenance, the condition of the pavement must be determined. This action can be completed manually or automatically. Currently, in Spain, these checks are manual, which is costly and slow due to the need for a human expert to identify the condition of these roads. Decision-making by an expert has drawbacks because the decision about the final state of a section of road is based on the subjective opinion of the individual based on perceptions that can be misleading, so erroneous information about it may be generated. The evolution of science and technology, together with the reduction in the cost of electronic component manufacturing, enable the investigation of new techniques and tools that objectively and automatically perform detection. Due to the above, universities and research centers are collaborating to obtain clear images of roads and detect any type of deterioration of a transport route.

At present, different techniques aim to detect the deterioration of road surfaces, for example, using a laser [2], vibration sensors [3], and images [4]. The development of techniques based on image processing has prompted research into machine learning methods capable of detecting different types of deterioration of road surfaces. In all the research using deep learning for image recognition, two problems must be solved: These techniques only work accurately if the configuration of the camera is dynamic, i.e., it adapts to the context and the light conditions of the environment, and these techniques always involve manipulating the pixels of the images. Therefore, this process becomes computationally complex when applied on computationally limited devices, especially where the performance and duration of the battery are critical factors [5]. Several investigations have been conducted to find a solution to these problems [6,7].

In all the investigations that offered a solution to detection through the use of pattern recognition techniques, an algorithm was constructed that is capable of image recognition together with the elimination of the problems that traditional deep learning solutions entail—the You Only Look Once (YOLO) algorithm. YOLO [8] is an algorithm that works in real time and on computationally limited devices, since only a single forward propagation through the neural network is necessary to determine a prediction. This algorithm has been evolving in its different versions, including YOLOv3 [9] and YOLOv4 [10], which are the latest stable versions of the algorithm. The difference between the versions is that YOLOv3 uses characteristic pyramid networks (FPN) for object detection, whereas YOLOv4 uses PANet as a method of aggregating parameters for different detection levels together with an increase in average precision (AP) and frames per second (FPS), a feature that makes the accuracy of YOLOv4 much higher than that of YOLOv3.

If we analyze the different methods of image collection to automate the process of capturing geolocated images, some solutions are based on the use of a vehicle with a camera close to the ground; however, this process is relatively slow because the vehicle must move at a very low speed so that the images obtained have a high degree of detail, since the movement of the vehicle affects the quality of the images. If the mapping area is large, several drivers are needed to reduce the time required to obtain the images. Therefore, the process becomes costly. Another solution is the use of satellite images; however, they are often outdated and the cost of accessing these images is high. Based on these shortcomings and trying to take advantage of the advances in recent years in the field of unmanned aerial vehicles (UAVs) and their low cost of acquisition, we used them as part of the data acquisition platform in the case study. UAVs contain different elements [11]: human, control, communication, recovery, and payload. These vehicles are used for surveillance, cartography, photogrammetry, fire detection, and the inspection of urban infrastructure, such as rooftops [12] or land [13].

In this study, we aimed to develop a platform capable of monitoring the state of roads and detecting different types of road damage to help coordinate the people and organizations involved in the identification process. To coordinate and communicate amongst all the entities involved in this task, the use of a multi-agent system (MAS) is proposed, which allows a dynamic reconfiguration of the system [14,15]. The multi-agent system distributes the resources and capacities to avoid problems that occur in centralized systems, such as bottlenecks or recurrent access to critical resources. The proposed multi-agent architecture increases the efficiency of the system by efficiently retrieving, filtering, and coordinating the information handled by the platform. The multi-agent system, which is used in the case study, is based on the use of the open-source platform PANGEA [16], allowing the different elements of the system to behave dynamically according to the requirements of the platform at any given moment. As a fundamental pillar in the identification of road damage, we customized the YOLOv4 algorithm. Notably, the machine learning models depend on the quality of the data used for their training; therefore, to obtain a robust model for this task, a new dataset was created for use by deep learning algorithms. Using a UAV is proposed to obtain the images automatically using a tracking road [17,18,19], thus minimizing the effort required for the sample collection process in terms of both cost and time. Through the proposed multi-agent architecture, coordination and communication between the different elements of the system are achieved so that the different classifiers that were tested are transparent to the end users.

The article is organized as follows: Section 2 provides an in-depth review of the current literature on crack and drone detection algorithms. Section 3 describes the architecture of the proposed system. The experiments and results are outlined and explained in Section 4. Finally, the conclusions are presented in Section 5. In addition, images of the pothole detection are provided in Appendix A.

## 2. Background

In this section, the different works in the different areas involved in this study are analyzed. This section is divided into different sub-sections to provide a more detailed analysis of each of the research areas. First, we begin by explaining the state-of-the-art technology used to capture the images from our dataset (drones). Then, we analyze the different methods and algorithms of image detection. We then focus on one of these image detection systems, YOLO, which is used in our framework, as well as some of the most used datasets. Finally, we refer to the framework in which we include the proposed system, the multi-agent system, highlighting its major advances and utilities.

### 2.1. Drones

The first step in determining the status of a pathway is to take images of it. To obtain images with good quality and perspective, we chose to use a drone. UAVs are becoming an important tool for surveyors, engineers, and scientists, as the number of cost-effective and easy-to-use systems is increasing rapidly. These platforms offer an attractive alternative for mapping small areas with centimeter resolution [20]. Drones are widely used today for tasks such as surveillance, monitoring, and data collection in buildings, infrastructure, and hazardous environments [12], detection of excessive vegetation growth [13], Internet of Things (IoT) systems [21], and in combination with virtual reality devices for control [22]. The main reason for why we chose a drone to recognize the terrain and obtain the data of the dataset was to acquire a large number of images in a short period of time with an acceptable resolution. Using these images, it is possible to associate the cracks in the recorded images with their real positions in the real world and to calculate their real sizes.

### 2.2. Pattern Detection in Images

Once we determined how to obtain the road image data, we focused on the area of object detection—in our case, cracks and potholes—in the road. In recent years, demand has been growing for analysis and recovery of large amounts of information from images on a network. The main purpose of image processing is to separate relevant information (foreground) from non-relevant information (background) [23].

Cracks are a common defect in structures, and they can cause serious structural failures. Surface crack detection using image processing has emerged as an important topic in the last 20 years [24]. Most studies have targeted crack detection for concrete surfaces [25,26], glass fiber composite laminates [27], solar cells [28], and different types of surfaces [29]. The use of the image detection techniques, which involve the detection of cracks and potholes in the captured image, allowed us to extract relevant information without pre-processing the image in a reasonable time with acceptable results.

### 2.3. YOLO

One of these systems used for image detection is YOLO. YOLO is an open-source application that allows the classification of images in real time.

We selected YOLO because it has been tested and used for the detection of cracks in different surfaces, such as defects in turbine propellers [30], cracks in cement surfaces [31,32], and in composite panels [33]. YOLO has previously been used for the detection of cracks in roads [34,35,36,37,38].

To select the appropriate version of YOLO, we compare the main versions of YOLO in Table 1, focusing on different aspects like the number of convolutional layers, activation function, calculation function, and the specific characteristics of each version.

Table 2 compares the mean average precision (mAP) and speed (FPS) for the PASCAL VOC2007 dataset made in [46]. The YOLOv2 version has the highest FPS, and YOLOv3 + SPP obtains the highest mAP.

To improve YOLO’s efficiency, it is used in combination with different algorithms and techniques, as well as different variants of the original YOLO model.

The variants include the YSODA model for the detection of small objects [30], the DC-YOLO model based on YOLOv3 [42], the Tiny-YOLO-V2 model, which has a lower computational cost, and pre-trained detection models, such as DarkNet [42]. The DarkNet model has pre-trained networks that are capable of recognizing and detecting cracks in pavement, which is why we chose to use it in our system.

For crack detection, there are two-phase and single-phase methods. Both have their disadvantages: The first is too slow (low FPS) and the second is less accurate than the two-phase method. Xia et al. [36] used the single-phase method, focusing on improving the accuracy of the method by incorporating a new detection pipe (cumulative feature module and a smoothed loss function). This update of the original algorithm was selected and added to our system because we needed high FPS and significantly higher accuracy.

### 2.4. Multi-Agent

We placed the image detection algorithm in a multi-agent system platform framework to control the drone movements and coordinate all the functions of the system. We selected the multi-agent system because it is an ideal platform for improving communication between agents due to their maneuverability and their ability to be deployed in a multitude of environments [47] due to the use of the RL and Q-learning algorithms. They are useful for tasks in different areas, both in commercial and industrial environments, such as search and rescue of people, sensorization, monitoring of the environment, surveillance, and recognition, as well as in other scientific fields [47,48].

For our purpose, we selected the MARLsystem, which performs general-purpose tasks in a coordinated and efficient manner [48]. After a detailed review of the literature, no reference or knowledge was found of a crack detection application that makes use of distributed technological capabilities adapted to the recognition of Spanish roads.

## 3. Proposed System

### 3.1. Proposed Architecture

The proposed architecture aims to provide a solution to the problem and can be adapted to the introduction of new functionality in different environments. An architecture to deal with the proposed problem must contain a series of well-defined characteristics to correctly operate. To this end, this research project was designed to focus on a multi-agent architecture that allows for the automation and detection of irregularities on roads by employing the detection of images captured by a drone. The main characteristics that led to the decision to use this type of architecture are its distributed architecture, the high level of communications, and the existence of message queues for information processing. A multi-agent architecture also allows a functionality used by one system to be replaced by another without having to modify the whole system, such as the image processing system or the type of database. A multi-agent system, in addition to the characteristics described above, is mainly characterized by each agent having a well-defined task and coordinating among themselves to achieve a common goal.

**Figure 1:**
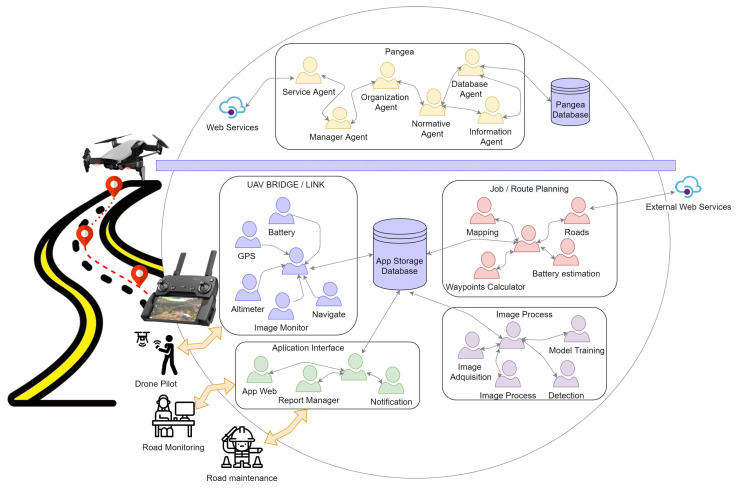
Proposed architecture using multi-agent system (MAS) PANGEA.

For the construction of multi-agent systems, several systems have already been developed that allow the process to be faster, including libraries such as SPADE, Python’s library [49], or more complex systems, such as JADE [50], PANGEA [15,51], and osBrain [52]. In this architecture, we chose to use the PANGEA multi-agent system as a starting point. This system allows the elements of the system to dynamically enter and leave the platform, thus allowing the specific demands of the system to be met. The MAS architectures have to perform services on demand, which means that each of the agents has to report on the services they have available and can offer to other entities within the architecture. PANGEA is based on organizational technology, which allows for visual representation and can be applied to any type of system, allowing for human interaction within the system. Finally, PANGEA can be distinguished from other systems by its rules engine that allows for the distribution of the computational load. Figure 1 shows the proposed architecture using MAS PANGEA, the different virtual organizations, and the main agents that form the architecture designed for this system.

The designed architecture is divided into different parts. There are two well-defined parts: The upper part of the image displays the minimum agents necessary for the functioning of the MAS PANGEA system; the lower part shows the virtual organizations upon which the case study was based.

The organizations in the system are detailed below.

Job/Route Planning Organization: This organization refers to the generation of the routes to be followed by the drones to capture the video of the roads. Its main functionality is based on transforming data between two coordinates into a safe route that the drone can follow to capture information. Communications between the agents of this organization and the central agent are bi-directional. This agent is responsible for requesting services from each of the agents, and they respond with data for each of the queries. This central agent is responsible for communicating with the other organizations for the exchange of information. The agents found in this organization are listed in Table 3.

Image Process Organization: This organization is responsible for processing images by applying deep-learning techniques. To achieve the objective of this organization, it has several agents that can be replaced by agents of the same characteristics that apply different techniques for labeling, training models, or detection. This architecture, which allows for exchanges of agents, makes it easy to conduct tests or apply new techniques without making changes to the whole system. The main agents that can be found in this organization are as follows:

- Image acquisition: Obtains the images from the source to perform pre-processing, training, or detection tasks.
- Image pre-process: Performs pre-processing of the images, such as color adjustment, scaling, and adaptation of each of the images to the inputs of the different models.
- Model training: Takes the tagged images as input and performs training with different deep learning algorithms.
- Detection: Uses the deep learning models trained by the training model agent to detect cracks or anomalies in the new images inserted into the system.

UAV Bridge/Link Organization: This organization refers to the application that is used as an interface for communication and information exchange between the drone and the platform. It contains several agents that obtain data from the drone sensors, such as the battery agent, the altimeter agent, or the image monitoring agent. In this organization, we also have a navigation agent that is in charge of the navigation of the drone. This agent uses the information from the other agents in the organization and the coordinates of the waypoints from the job/route planning organization to make the drone reach the indicated coordinates. The global positioning system (GPS) agent, apart from being useful for navigation, is crucial for the whole system, since it allows tagging of the locations of the photos and, thereby, locating where crack repairs are necessary.

Application Interface Organization: This organization can adapt the generated information to the application layer. This organization is used as an interface; the applications that interact with this interface can interact directly with the system. That is, the organization is responsible for converting the system’s raw data to human-readable data. In this particular case, it is used for the user in charge of monitoring and deciding which roads are to be inspected, the applications of the workers responsible for carrying out repairs on these roads, the generation of reports, and sending notifications.

PANGEA Multi-Agent System Organization: This organization is composed of the minimum agents necessary for PANGEA’s operation. The objective of this organization is to manage virtual organizations and the agents in each of them. The agents of this organization include the following:

- Database Agent: The only agent with database access privileges, it stores the information present within the organization. This agent is also in charge of performing backups and ensuring consistency of information.
- Information Agent: Manages the services within the virtual organization by making the services of each of the agents available to the other agents. When an agent joins the system, it indicates to this agent the services it provides so that when another agent needs to use that service, it makes a query to this agent.
- Normative Agent: Responsible for imposing and ensuring that the rules comply with the communications they establish between agents.
- Service Agent: Aims to distribute functionality through web services. It is used as a gateway that allows the communication of external services with the organization’s agents. These web services allow the easy construction of external agents in different programming languages.
- Manager Agent: Responsible for checking the status of the system periodically. It is in charge of detecting if there is any overloaded functionality and any possible failures in the agents of the different organizations.
- Organization Agent: Responsible for the verification of all the operations of the virtual organizations, checking the security and the load balancing, and offering encryption of the most important agents.

The system uses two different databases for its operation: The PANGEA Database is used by the PANGEA organization to store the available agents and the services available in each of them. The APP Storage Database contains information about the specific system. In this case, this includes the images obtained by the drone, their locations, the next inspections to be performed, routes, etc.

In the proposed architecture, three agents external to the system were designed. These represent the users who interact with the system. Firstly, Road Monitoring is the main agent in charge of defining the inspections of the roads to be carried out, labeling the images, and supervising of the results. The second agent of the system is the Drone Pilot, which is in charge of conducting road inspections. Its main task is to supervise the flying of the drone in case there is any incident and the drone needs to be controlled manually. Finally, the Road Maintenance agent represents the person or company responsible for completing road maintenance and resolving incidents.

The architecture is based on different modules and each one is specialized in a different task or objective. The challenge of this type of architecture is focused on simplifying communication between nodes or agents and allowing them to be decoupled from the system so that they can be replaced by others with similar characteristics or services. To this end, when the system is initiated, we have a set of agents from the PANGEA organization that are responsible for displaying the available services and coordinating agents.

**Figure 2:**
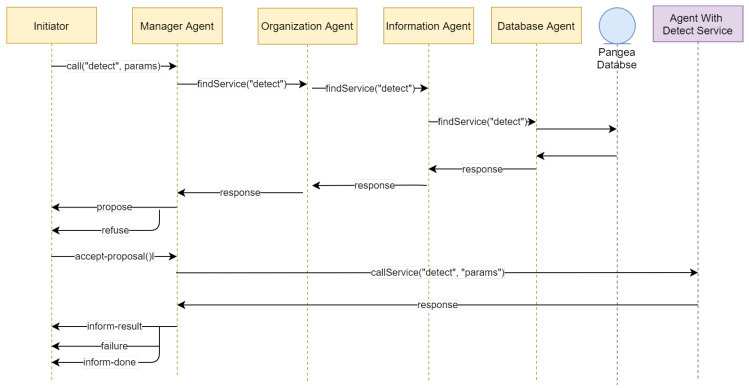
Image call service.

The protocol used in this architecture to achieve the objectives is the contract-net protocol, where an external agent can search, find, and execute a required service. To do this, the agent that requires a service sends a message to the Agent Manager indicating the type of service required with the name of the service and the parameters for that service. This agent, with the help of the Organization Agent, Information Agent, and Database Agent, responds with a list of available agents that have the services and resources to carry out the required service. The agent accepts the proposal of the Agent Manager by choosing an agent from the list to carry out the service. An example of requiring a detection service is provided in Figure 2.

The architecture in the case study based on PANGEA allows the dynamic integration of new functionality without affecting the other parts of the system. The second reason why PANGEA was chosen is its user license, as it is open source can be used for commercial purposes.

### 3.2. Proposed Method

We evaluated the different forms of malfunctions and cracks in roads using deep learning for detection. The aerial system consisted of a DJI Mavic Air 2 quadcopter (DJI, Shenzhen, China), with a maximum flight time of 34 min, combining a 4K digital camera and location information, which were used for aerial imaging. The camera mounted on the UAV had a 24 mm lens with a field of view (FOV) of 84 degrees and a resolution of 4000 × 3000, and it was capable of shooting 48-megapixel photos; the camera was three-axis stabilized by its gimbal drone https://www.dji.com.

**Figure 3:**
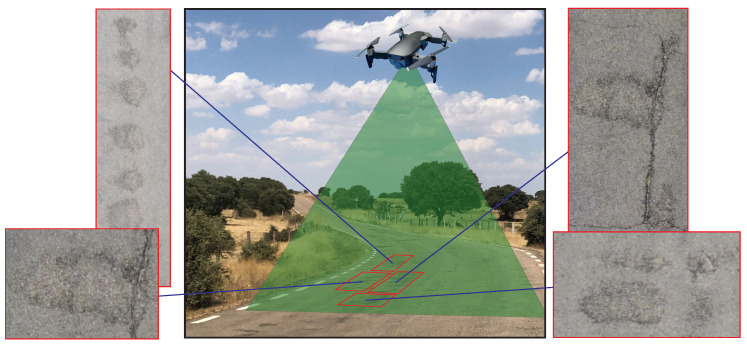
Image data acquisition.

The image data used for model training and testing of deep neural networks were collected using the conventional UAV camera, since its resolution capacity was 3840 × 2160 pixels at a distance of 60 m from the ground. According to some authors [53], during this training process, images can be used from third parties with different environments but following the same process. The general idea of this proposed solution is illustrated in Figure 3.

During the capture process, videos were created. From them, frames were made for use in training. A total of 568 high-resolution images were generated, which were then classified and labeled as potholes and cracks. Then, the data from the labeled images were used to train and evaluate the convolutional neural net; the dataset used in this work is detailed in this section.

**Figure 4:**
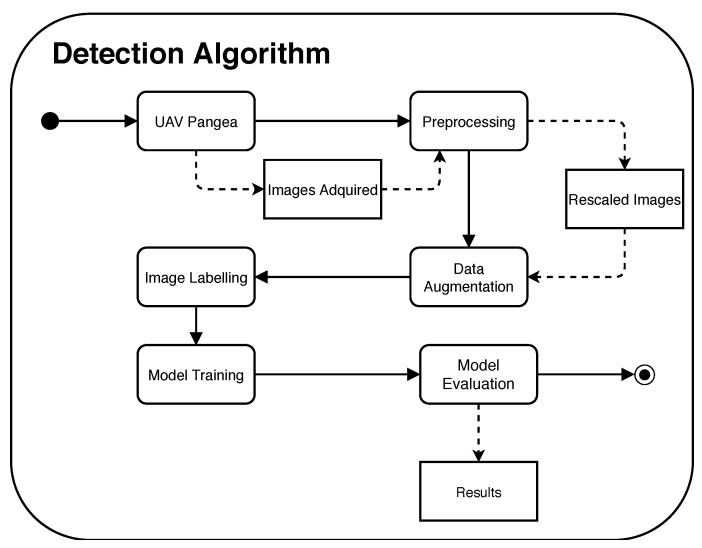
Block diagram of the drone detection solution.

We first prepared of research data, followed by annotation and labeling. The prepared dataset was divided into training and testing sets. The labeled training data were used to build a model using the YOLOv4 architecture. The result of the modeling phase was a model, also called weight. To evaluate the model’s performance, we detected the cracks found in the roads that were saved in the test data. The proposed method is shown in Figure 4.

#### 3.2.1. Annotation and Labeling

The classification process is a manual process and must be performed by a person who knows how to differentiate whether the object in question is in the image and where the object is located. For this process, we used LabelImg software [54]. This graphic tool allows the annotation of images and delimitation of boxes of labeled objects in images. During this process, more than one object can be found, and each one must be delimited.

In this process of marking, we completed the annotations on all 568 images acquired. The information was stored in text, starting with the identification or annotation, differentiating potholes and cracks. In this case, a Class ID, the central position of the bounding box (x and y), and width and height of the bounding box (w and h) were stored. The Class ID is an integer value, starting at 0, and the bounding box information is a decimal format on a scale of 0–1. Each image in .jpg format has a file in .txt format with information about the holes, as shown in Equation (1).

(1) $$\begin{array}{c} 0\;0.365625\;0.745370\;0.020313\;0.055556\\ 0\;0.665755\;0.531481\;0.048698\;0.053704 \end{array}$$

#### 3.2.2. Model Training

This work was based on the YOLOv4-tiny model, which follows the principle of prediction of coordinates like YOLOv2 and YOLOv3. It is possible to multi-classify in YOLOv4 instead of classifying only one class, as in the older versions. YOLOv4 adopts a cross-loss entropy function instead of the multi-class loss function.

The network was configured to detect two classes. The filter number must be configured directly in the layer of the convolutional network. Thus, the formula used to apply the filter number is represented by Equation (2).

(2) Filters=(Classes+5)×3

**Figure 5:**
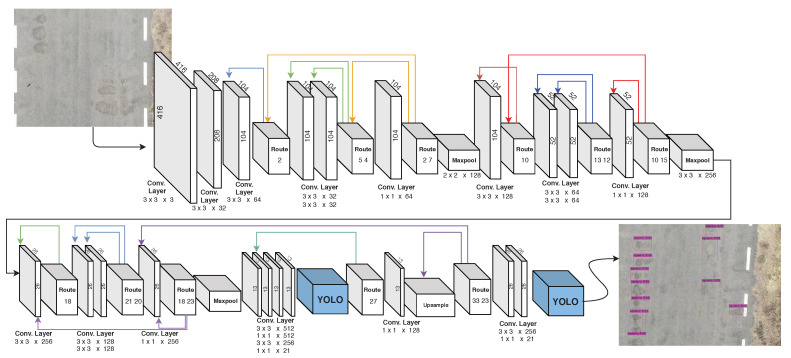
The network structure of the YOLOv4 pothole detection model.

This number of filters must be replaced in the three convolutional layers before each YOLO layer, which only has to be the last convolution before each of the YOLO layers. In the model used in this work, the two last layers before each YOLO instance have 21 filters each. The networks used and configured are presented in Figure 5, highlighting the YOLO instances in blue.

By using this model in a graphics processing unit (GPU), YOLOv4 can be used with real-time images [55]. However, in devices with low processing capacity, such as the Nvidia Jetson, the conventional YOLOv4 algorithm works slowly. As an alternative, the YOLOv4-tiny network can be used, which satisfies the requirements in real time based on limited hardware resources. Therefore, we chose to use the YOLOv4-tiny algorithm. The structure of the YOLOv4-tiny network used is shown in Table 4.

For the training, we used the 1362 images present in the dataset. An image could be reproduced several times, increasing the training of our model. During the pre-processing stage, we manually detected and recognized the holes in the road, as explained in Section 3.2.1. The model training environment is composed of the DarkNet network. This neural network structure is written in C and CUDA language and can be executed directly on the GPU. It is installed depending on the GPU; we used it with Google Colab, which allowed us to perform calculations on a Tesla K80 GPU with 12 GB of memory.

## 4. Results

To evaluate our system, we first considered quantitative aspects, comparing the labeled dataset images with the final images classified by the algorithm. Next, for the qualitative evaluation, a series of applications were constructed to allow the user to carry out, in a centralized and unified way, all the tasks of our system, from the definition and follow-up of routes to the visualization and verification of the results.

### 4.1. Dataset

According to [34], the main objective in creating better roads is to avoid holes and cracks in the track, which requires precise diagnosis and differentiation between the types of problems that can be found in the pavement.

**Figure 6:**
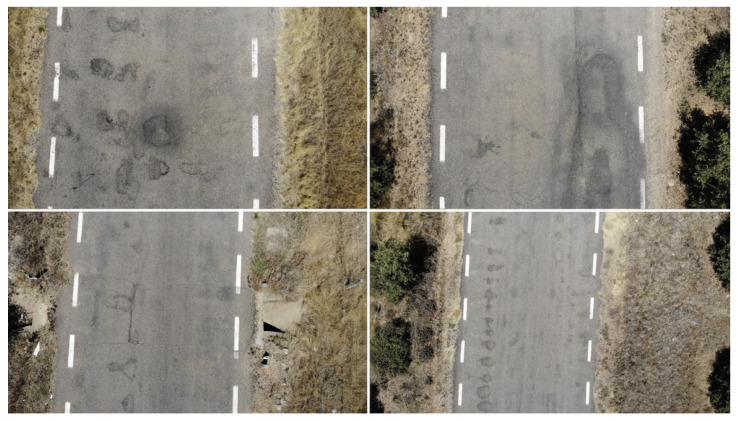
Dataset.

In the bibliographical research, we identified several datasets of holes and cracks in asphalt, but the datasets were not adequate for the proposed method of using a drone in a multi-agent system to capture pictures at a safe distance from the road. Therefore, we created of a new set of data to represent the situation of Spanish roads. A total of 600 photos were taken at a resolution of 3840 × 2160 pixels. The images were resized to 1200 × 900 pixels. Some images of the dataset are demonstrated in Figure 6.

After labeling, the dataset contained a total of 568 labeled images. During the pre-processing stage, adjustments were made to the image orientation and resizing, as mentioned above. For each image in the set, different versions of the image were created using magnification techniques. The images were zoomed in and out, ranging from 0% to 15%. This process was repeated to increase the data set size from N to 2N, repeating only the images that contained defects in the track.

**Figure 7:**
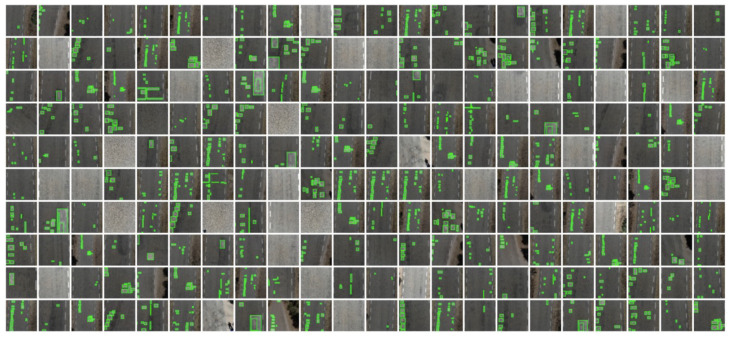
Dataset.

The total number of images in the dataset was 1362 images. Of these images, 70% were for training, 20% for validation, and 10% for testing the effectiveness of the trained model. The dataset is composed of the images and their respective labels. An application (https://roboflow.ai) was used to generate the dataset, according to Figure 7.

The dataset used for the validation of the results of this scientific article has been published at https://github.com/luisaugustos/Pothole-Recognition for verifying the results or for testing new algorithms by the community.

For the quantitative evaluation, we used the following metrics: accuracy, i.e., the relationship between true positives (TPs) and true positives (TPs) along with false positives (FPs) (Equation (3)); recall, which is the probability that an image is classified as positive and the relationship between the TPs and the TPs together with the false negatives (FNs) (Equation (4)); and F1, which is combination of the two previous metrics (Equation (5)). We classified the speed measured in frames per second (FPS); the mean average precision (mAP), calculated by the precision and recall curve; and the intersection over union (IoU), which is the overlapping area between the area found the image and the detected area. Lastly, we used the kappa metric (Equation (6)), which is the relationship between the relative observed agreement among raters Pr(a), and the hypothetical probability of chance agreement Pr(e), which is used to measure inter-rater reliability for qualitative items.

(3) $$Precision=\frac{TP}{TP+FP}$$

(4) $$Recall=\frac{TP}{TP+FN}$$

(5) $$F1=2\cdot \frac{Precision\cdot Recall}{Precision+Recall}$$

(6) $$Kappa=\frac{Pr(a)-Pr(e)}{1-Pr(e)}$$

**Figure 8:**
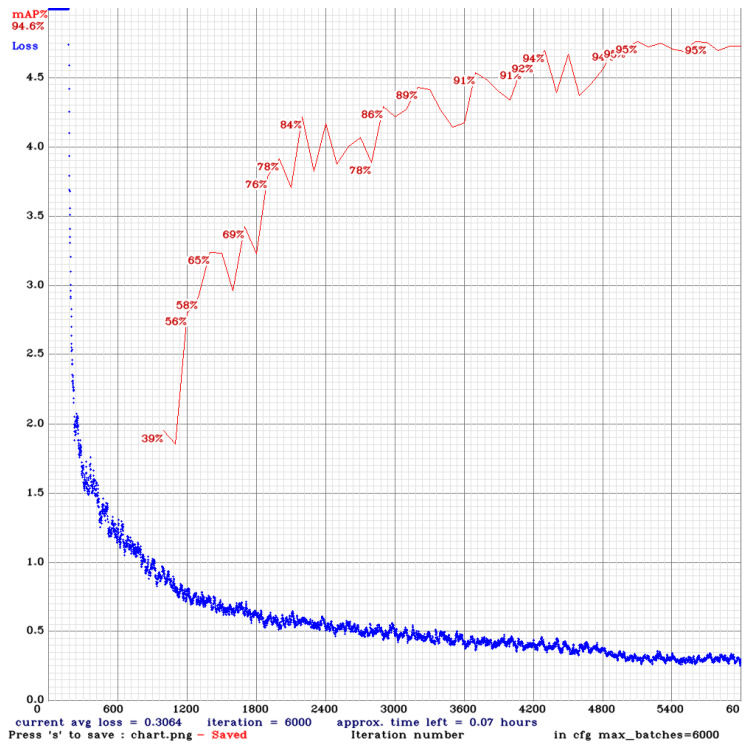
The result using 608 × 608 images with 6000 batches.

The training process of the model was evaluated in stages, alternating between iterations and image resolution. The first stage was used for YOLOv4-tiny using a pre-trained weight model, altering the convolution layers as needed. Every 1000 iterations during the training process, the model was stored; the result of the success of each of the values is shown in Figure 8. In the image, the value reached 6000 iterations and stabilized with an average success value of 94.6%.

### 4.2. Detection

YOLOv4 has four data augmentation parameters provided by the author that were also used to generate more training samples by rotating the angle and adjusting the saturation, exposure, and hue of the images. YOLOv4 uses a new method of data enhancement, which was explained earlier in Section 2. The speed of image detection with YOLO initially averages 45 FPS. Its biggest failure is inaccuracy with small objects in the image.

**Figure 9:**
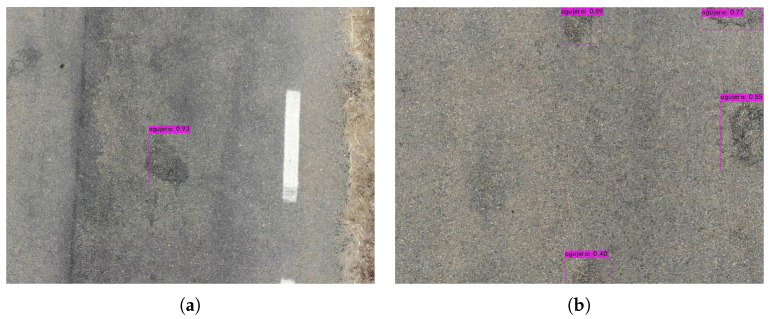
Detection results with zoom. (**a**) Single detection. (**b**) Multiple detection.

**Figure 10:**
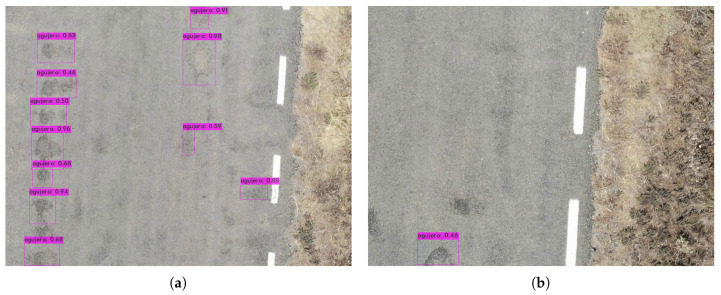
Detection results in a large number of potholes. (**a**) Multiple detection. (**b**) Single pothole.

**Figure 11:**
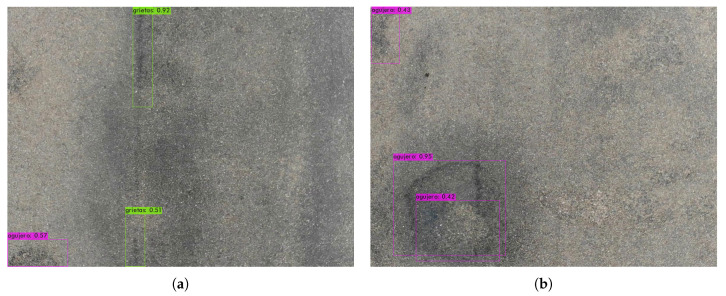
Crack detection and a detection of a pothole inside other. (**a**) Detection of a crack in the track. (**b**) Pothole inside another.

The bounding box (bbox) method, for each grid cell, predicts B (bbox) and C probabilities of being one of the trained classes. A bbox prediction has five components: (x, y, w, h, and confidence). The coordinates (x, y) represent the center of the bbox relative to the location of the cell. If the center of a bbox is not in a cell, it will not be responsible for it and will not represent it. Cells only have a reference to objects whose center falls inside them. These coordinates are normalized to [0, 1]. The dimensions of the bbox (w, h) are also normalized to [0, 1] relative to the image size. Figure 9, Figure 10 and Figure 11 depicts the result in the detection of road defects. In addition, results in Appendix A and a video demonstration added in Supplementary Materials.

In experiment 1 (Table 5), concerning the label that classifies the potholes, we observed an accuracy of 96.25% and an AP of 98.46%. For the classification of the cracks, we obtained slightly worse accuracy and AP than for potholes, at 90.38% and 90.89%, respectively. This decrease occurred due to the lower number of images of cracks in the dataset. For all the images, the precision was 95.70%, the AP was at 94.67%, and the prediction time was 5.53 ms. We obtained a kappa metric of 0.73, which represents a high concordance.

In experiment 2 (Table 6), we obtained similar results to those in experiment 1. In the classification of the potholes, we obtained a precision of 96.54% and an AP of 98.45%; however, in the classification of the cracks, the precision was higher than in experiment 1, obtaining a similar AP of 92.00% and 90.68%, respectively. In all the images, the precision was 96.13%, the AP was 94.56%, and the prediction time was 5.52 ms. We obtained a kappa metric of 0.72, which represents high concordance, as in the first experiment.

To achieve complete functioning of the system, three different applications were developed, each with a well-defined objective, which can used by users with different roles within the system.

### 4.3. Application

**Figure 12:**
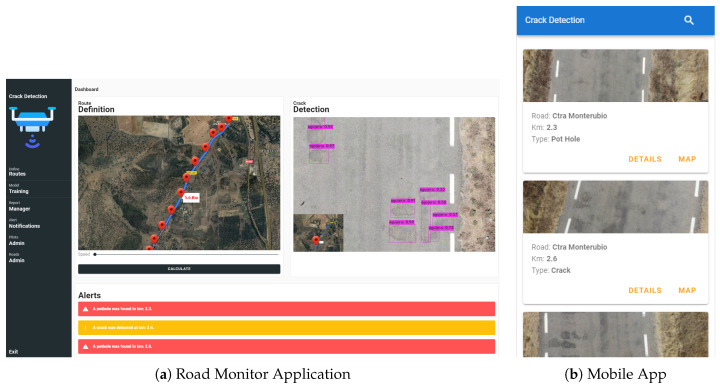
Application details.

The first application in Figure 12a refers to the application used by the Road Monitor. In this application, the user is responsible for defining the routes where inspections should be performed, preparing the data for training, and validating the data of the detection output for the generation of reports and worksheets for the operators in charge of performing road maintenance. Although the system allows automatic operation, we decided to use expert supervision to avoid possible failures, identify the cracks or holes that need intervention, and assign these tasks to the operators. This reclassification will allow feedback on the system to improve the algorithm in the future.

The second application we developed is the Road Maintenance users’ application, which is shown in Figure 12b. In this application, the user has a list of incidents, for which details, the photo, and the location of the incident are provided to enable its repair.

The last application is that used by the drone pilot. This application specifically uses the DJI SDK, since the drone used for the case study is of this brand. In this application, a list of tasks to be performed is provided and used by the pilot to know where the inspections are required and to perform real-time monitoring of the route followed by the drone during the inspection. This application takes the route coordinates calculated in the Road Monitor application to automatically create a route.

The main advantage offered by the development of the set of applications compared to the use of applications that currently exist on the market is a complete system from the definition of routes, to inspection, to the monitoring of results.

## 5. Conclusions

In this work, we designed a platform capable of detecting damage on transport routes using drones and a multi-agent architecture. At the level of detection of anomalies, accurate results were obtained, since the proposed system enabled the platform to achieve precision higher than 95%. Despite the different crack detection techniques that were tested and analyzed during the literature review phase, none of them produced a result higher than 47%. Notably, one of the key factors producing this low performance was that the datasets found in different articles corresponded to non-European roads or that cracks were too large, so they were not similar to the small cracks and potholes that were detected in this work. The dataset for the verification of results in this work was developed on a regional road in the province of Salamanca. The imaging was performed according to the current legislation for the flight of drones in public locations. No variation was observed in the results obtained by the predictive algorithm between images taken at 70 m and at 90 m. The results were invariant regardless of the speed of the drone, which was 15–25 km/h during the tests.

The findings demonstrated how the procedures currently carried out by companies and regional governments responsible for road maintenance can be improved. Our method saves time, costs, and labor by establishing a more objective method for determining the road areas in need of repair as soon as possible.

The design of the solution proposed in the case study and based on virtual organizations of agents allows the testing of different techniques and the definition of parameters that are suitable for the user’s final application, that is, without the final user detecting any kind of change during the reprogramming. The PANGEA-based multi-agent architecture allows for parallelization of work according to the requirements of the platform at any given time, thus adapting to the needs of the context. The advantages of using an MAS include facilitating the development of case studies and ensuring compatibility between the different entities that compose the platform. The communication of the different agents implemented by means of the RFC 1459 Internet Relay Chat Protocol allows the optimization of the energy consumption necessary for communication, thus optimizing the battery of the drone as much as possible during the flight. To allow the scientific community to carry out research, both the pre-trained model and the datasets have been made public in a repository.

As future lines of this work, we plan to work on the coordination and distribution of works using drones with the main goal of distributing or subdividing a sampling area among several UAVs with different characteristics, such as weight, speed, and flight time. Due to this, we are currently studying different techniques to optimize tasks and routes based on battery life, altitude, and distance from the mapping zone to the initial point of takeoff using ardupilot as a flight controller. We also plan to continue our research by incorporating new 3D cameras or a LiDAR sensor in this drone or in a custom-made drone that allows knowledge of the distance from the drone to the road to calculate the size of the cracks or holes. Although we have started tests using techniques based on drone examples, no important results are yet available for dissemination.

## Figures and Tables

**Table 1:** You Only Look Once (YOLO) versions.

Version	Article	Features
YOLOv1	[4,30,31,34,35,39,40]	26 layers, SoftMax activation function, sum-squared risk calculation function, may not detect objects too close together, non-maximal suppression to eliminate duplicates.
YOLOv2	[35,41].	30 layers, function of calculation of the risk of mean square error, requires fewer training times, batch normalization in the layers (an increase in the mean average precision (mAP)).
YOLOv3	[37,42,43,44,45].	106 layers, multi-label sorting, SoftMax activation function with independent logistic sorters, binary cross-entropy loss calculation function for each label, small object enhancement.
YOLOv4	[10].	53 layers, FPN for greater accuracy, SAM allows focus on a specific part of the image, SPP detects object deformations.

**Table 2:** mAP and speed comparison.

Method	mAP (%)	Speed (fps)
Faster RCNN	76.4	5.0
SSD	74.3	46.0
DSSD 321	78.6	9.5
STDN	78.1	40.3
YOLO	63.4	45.0
YOLOv2	76.8	67.0
YOLOv3	79.3	37.9
DC-SPP-YOLO	78.4	55.7
YOLOv3(DC)+SPP	79.7	37.0

**Table 3:** Agents of the job/route planning organization.

Agent	Description
Mapping	Allows iteration with maps to identify the existence of high-voltage cables in the flying area, distances to airports, etc.
Waypoint Calculator	Calculates optimal waypoints from route data.
Roads	Obtains a route from a road indicating two coordinates. This agent connects to external service APIs. One can use the services of Google or Open-Street Maps to obtain this information.
Battery Estimation	Estimates the battery life for the realization of a route.

**Table 4:** Network structure of YOLOv4-tiny.

Layer	Type	Filters	Size/Stride	Input	Output
0	Convolutional	32	3 × 3/2	416 × 416 × 3	208 × 208 × 32
1	Convolutional	64	3 × 3/2	208 × 208 × 32	104 × 104 × 64
2	Convolutional	64	3 × 3/1	104 × 104 × 64	104 × 104 × 64
3	Route 2				
4	Convolutional	32	3 × 3/1	104 × 104 × 32	104 × 104 × 32
5	Convolutional	32	3 × 3/1	104 × 104 × 32	104 × 104 × 32
6	Route 5 4				
7	Convolutional	64	1 × 1/1	104 × 104 × 64	104 × 104 × 64
8	Route 2 7				
9	Maxpool		2 × 2/ 2	104 ×104 × 128	52 × 52 × 128
10	Convolutional	128	3 × 3/1	52 × 52 × 128	52 × 52 × 128
11	Route 10				
12	Convolutional	64	3 × 3/1	52 × 52 × 64	52 × 52 × 64
13	Convolutional	64	3 × 3/1	52 × 52 × 64	52 × 52 × 64
14	Route 13 12				
15	Convolutional	128	1 × 1/1	52 × 52 × 128	52 × 52 × 128
16	Route 10 15				
17	Maxpool		2 × 2/ 2	52 × 52×256	26 × 26 × 256
18	Convolutional	256	3 × 3/1	26 × 26 × 256	26 × 26 × 256
19	Route 18				
20	Convolutional	128	3 × 3/1	26 × 26 × 128	26 × 26 × 128
21	Convolutional	128	3 × 3/1	26 × 26 × 128	26 × 26 × 128
22	Route 21 20				
23	Convolutional	256	1 ×1/1	26 × 26 × 256	26 × 26 ×256
24	Route 18 23				
25	Maxpool		2 × 2/2	26 × 26 ×512	13 × 13 × 512
26	Convolutional	512	3 × 3/1	13 × 13 × 512	13 × 13 × 512
27	Convolutional	256	1 × 1/1	13 × 13 × 512	13 × 13 × 256
28	Convolutional	512	3 × 3/1	13 × 13 × 256	13 × 13 × 512
29	Convolutional	21	1 ×1/1	13 × 13 × 512	13 × 13 × 21
30	YOLO				
31	Route 27				
32	Convolutional	128	1 × 1/1	13 × 13 × 256	13 × 13 × 128
33	Upsample		2x	13 × 13 × 128	26 × 26 × 128
34	Route 33 23				
35	Convolutional	256	3 × 3/1	26 × 26 × 384	26 × 26 × 256
36	Convolutional	21	1 × 1/1	26 × 26 × 256	26 × 26 × 21
37	YOLO				

**Table 5:** Obtained results.

Label	TP	FP	FN	Precision (%)	AP (%)	Kappa	Prediction Time (ms)
Pothole	977	38	-	96.25	98.46	-	-
Crack	94	10	-	90.38	90.89	-	-
Total	1070	48	62	95.70	94.67	0.73	5.53

**Table 6:** Obtained results.

Label	TP	FP	FN	Precision (%)	AP (%)	Kappa	Prediction Time (ms)
Pothole	977	35	-	96.54	98.45	-	-
Crack	92	8	-	92.00	90.68	-	-
Total	1069	43	64	96.13	94.56	0.72	5.52

**Figure A1:**
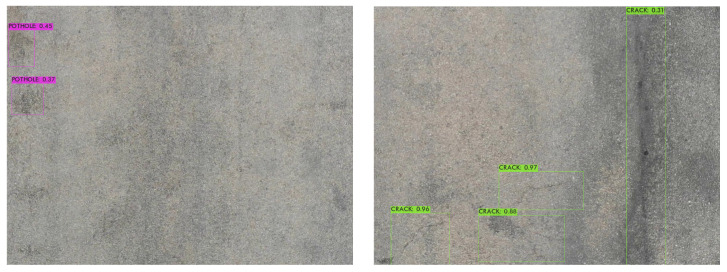
Detection results.

**Figure A2:**
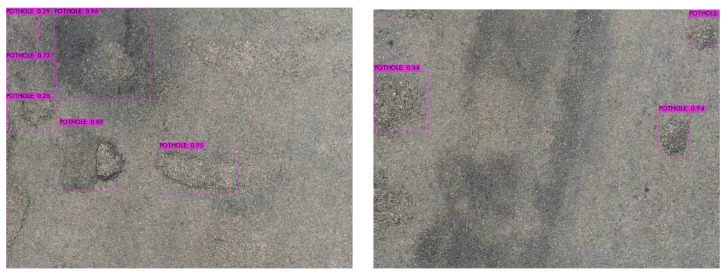
Detection results.

**Figure A3:**
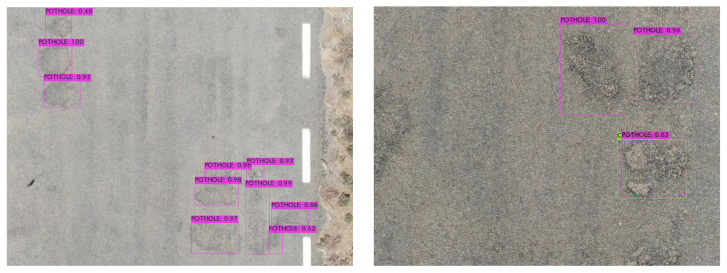
Detection results.

**Figure A4:**
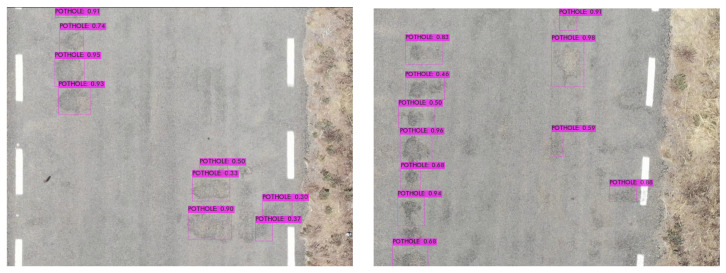
Detection results.

**Figure A5:**
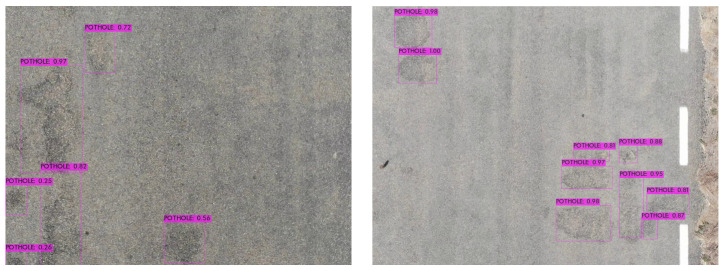
Detection results.

## Supplementary Materials

The following are available online at https://www.mdpi.com/1424-8220/20/21/6205/s1.

## Abbreviations

The following abbreviations are used in this manuscript:

AEC	Spanish Road Association
AP	Average Precision
FPS	Frames Per Second
FPN	Feature Pyramid Network
GPS	Global Positioning System
IoT	Internet of Things
JADE	Java Agent Development Framework
mAP	Mean Average Precision
MAS	Multi-Agent System
MARL	Multi-Agent Reinforcement Learning
YOLO	You Only Look Once
PANGEA	Platform for Automatic coNstruction of orGanizations of intElligent Agents
UAV	Unmanned Aerial Vehicle
FPN	Feature Pyramid Network
SAM	Spatial Attention Module
SPP	Spatial Pyramid Pooling
RL	Reinforcement Learning
PANet	Prototype Alignment Network
SPADE	Smart Python Agent Development Environment
